# Physical Activity and Sedentary Behaviors in Primary School Children in Saudi Arabia during the COVID-19 Pandemic: Association with Parents’ Behaviors

**DOI:** 10.3390/ijerph192013304

**Published:** 2022-10-15

**Authors:** Osama Aljuhani, Rola Alsuwailem, Abdulelah Al-Salawi, Gavin Sandercock

**Affiliations:** 1 Department of Physical Education, College of Sport Sciences and Physical Activity, King Saud University, Riyadh 4545, Saudi Arabia; 2 Department of Exercise Physiology, College of Sport Sciences and Physical Activity, King Saud University, Riyadh 4545, Saudi Arabia; 3 Department of Sport and Recreation Management, College of Sport Sciences and Physical Activity, King Saud University, Riyadh 4545, Saudi Arabia; 4 School of Sport, Rehabilitation and Exercise Sciences, University of Essex, Colchester CO4 3SQ, UK

**Keywords:** physical activity, accelerometer, Saudi Arabia, school children

## Abstract

During the COVID-19 pandemic, a few studies used accelerometers to assess physical activity (PA) and sedentary behavior in the family context. This study aimed to assess children and parents’ moderate and vigorous physical activity (MVPA) and sedentary time, as well as their relationship in MVPA and sedentary time. Data were collected from 30 parent–child dyads during the COVID-pandemic for seven days, using a hip-worn accelerometer. Children and parents engaged in 65.6 and 34.6 min/day in MVPA and 442.2 and 427.9 min/day sedentary, respectively. There was no evidence of gender difference in MVPA and sedentary between boys and girls. Male parent spent more time in MVPA than female parents. A total of 50% of children and 53.3% of parents met the recommended PA. Children’s MVPA and sedentary time were both correlated with that of their parents. Adjusted linear regression showed that only child MVPA was negatively associated with their parents’ MVPA. There is evidence that multi-level interventions involving parents and children are more effective than interventions focusing on a single group. This study also provides evidence to support the link between MVPA and sedentary time between parents and children. Generalization of the findings is difficult due to the bias of self-selection sample.

## 1. Introduction

In children and adolescents, increasing physical activity (PA) and reducing inactive time are linked to a variety of health benefits. Higher levels of regular PA and less time allotted for sedentary behavior during childhood and adolescence have been related to cardiometabolic health, skeletal health, cardiorespiratory fitness, academic performance, executive functions, mental health, and quality of life [1,2,3,4,5]. Therefore, to promote health, the Saudi Ministry of Health recommends that children and adolescents aged 6–17 years should engage in at least 60 min of moderate-to-vigorous intensity physical activity (MVPA) per day, and adults should accumulate 150 min of moderate intensity physical activity or 75 min of vigorous intensity physical activity per week [6]. However, previous evidence showed a reduction in Saudi children and adolescents’ PA levels, and 92% of Saudi adolescents aged 13–14 did not meet the recommended 60 min of MVPA each day [7]. Accelerometer data also showed that Saudi adolescent boys spent only 50 min of MVPA on a daily basis [8]. Among children, self-reported data showed that 59% of boys and 40% girls were classified as active, and 71% of boys and girls spent more than 2 h per day sedentary [9]. 

In March 2020, the World Health Organization (WHO) declared a severe health crisis (COVID-19) caused by new coronaviruses. Governments worldwide have implemented strict strategies to prevent the spread and effects of the virus on their citizens. On the 2 March 2020, when the first case of COVID-19 was confirmed in Saudi Arabia, the government implemented a range of strategies, such as closing schools and universities, travel restrictions, closing sports clubs, closing restaurants, closing mosques for prayers, and restrictions on attending government and private workplaces. Later, on the 9 March 2020, the first lockdown was announced and lifted on the 21 June 2020. However, strict measures, such as social distancing and closure of schools, have continued. These restrictions during the COVID-19 pandemic may have a negative effect on children’s PA and sedentary behaviors [10]. 

The majority of previous studies have reported a reduction in children and adults’ PA, and an increase in sedentary time during the COVID-19 pandemic. For example, a cross-sectional study using interviews showed a significant decline in PA in 52% of Saudi children and adolescents (*n* = 452) [11]. Additionally, a parent-based survey study reported a negative effect of the COVID-19 pandemic on Saudi primary school children [12]. In their study, Alanazi et al. [12] reported that only 35.7% of the study sample (*n* = 1021), between October and November 2020, met the recommended 60 min of MVPA per day, and only 15.2% spent 2 h or less in sedentary behavior. Internationally, a recent review also showed that 57 of 84 included studies reported a reduction between 10.8 and 91 min/day in PA levels in children and adolescents [13]. According to another systematic review and meta-analysis during the COVID-19 pandemic children’s inactive time increased by 159 min per day [14]. However, not all previous studies have reported a negative impact of the COVID-19 pandemic on children’s PA. Few studies have found that PA levels in children and adolescents increased during the COVID-19 pandemic compared with pre-pandemic levels [15,16,17]. Among Saudi adults, self-reported data showed that 52% of the study sample (*n* = 2255) reported a decrease in their daily PA during the COVID-19 pandemic [18]. Evidence also suggests an increase in adults’ sedentary time by 24% during the COVID-19 pandemic compared with pre-pandemic [19]. Inversely, no changes in Saudi adults’ PA levels were also reported from before to during the COVID-19 pandemic [20]. However, studies that assessed PA and sedentary behaviors in Saudi children and adults are limited by using subjective measures. Therefore, the influence of recall bias on the results can be presence. For example, existing evidence of PA and sedentary behavior from previous studies in Saudi Arabia relies on the parents’ awareness of their child’s PA and sedentary behaviors. Parents were found to incorrectly classify their children’s PA and sedentary behaviors [21]. In adults, evidence suggests that the self-report method may overestimate PA [22] and underestimate sedentary time [23]. Thus, more accurate estimation of PA and sedentary behavior during the pandemic period may be essential for PA promotion among children. However, with high sedentary time and low levels of PA activity reported during the pandemic, exploring factors that might link to children’s PA levels and sedentary behavior is essential for promoting their health.

Under typical living conditions, family factors such as parents’ behaviors and presence have been demonstrated to be major predictors of young children’s behaviors [24]. For example, a positive correlation has been found between children’s PA and parents’ role modeling [25,26] and co-participation in PA with children [24,27,28]. It was also found that adding 20 min to a parent’s MVPA resulted in a 5–10 min increase in children’s MVPA [29]. In addition to PA, a positive relationship was found between parents’ sedentary behavior and their children’s sedentary behavior [24]. Parental factors such as being younger and active are significantly associated with children’s PA and sedentary time during the COVID-19 pandemic [30]. In their survey-based study, Moore et al. (2020) found that Canadian children had less decline in PA if they lived with younger parents, and parental PA levels were associated with more child PA levels [30]. However, for better understanding of the influence of the COVID-19 pandemic on the association between children’s PA and sedentary time with parent behaviors using objective methods such as accelerometers is warranted. Among 22 Arab countries, no single study was found to objectively assess children and adults’ PA and sedentary behaviors during the COVID-19 pandemic nor to examine the association between parent and child PA and sedentary time. Thus, this study aimed to determine the amount of time Saudi children spent in PA and sedentary during the COVID-19 outbreak. Additionally, this study aimed to examine the relationship between children’s PA and sedentary behaviors, and their parents’ PA and sedentary behaviors. We hypothesized that children and their parents accumulate lower time in MVPA and higher time sedentary. Moreover, we also hypothesized that children’s MVPA and sedentary time are significantly associated with their parents, MVPA and sedentary time. 

## 2. Materials and Methods

### 2.1. Participants and Family Characteristics

Cross-sectional data were collected from parents and their children from a local community in Riyadh, Saudi Arabia. In this study, participants were invited to join the study by posting an announcement through social media and the university community. A convenience sample of 37 parent–child dyads agreed to participate in the current study. To be eligible for participation, only participants living in one home together were included. Additional inclusion criteria were to have at least one child between 7 and 12 years of age and at least one parent between 18 and 64 years of age. Exclusion criteria included major health conditions affecting mobility. Before conducting the study, consent forms explaining participation requirements were obtained from the students and their parents. The King Saud University Ethical Review Committee from King Saud University approved the study ethics (ethics code: KSU-HE-20-256).

We used G*Power statistical power analysis software (version 3.1.9.4) to calculate the required sample size, which is 40. However, due to data exclusion and COVID-19 restrictions, final sample size used was 30. Seven child–parent datasets were excluded from data analysis because they did not meet the inclusion criteria of wearing the accelerometer for at least 600 min/day and for a least 3 days per week. This may increase the risk of attrition bias between excluded and included groups. However, the preliminary analyses revealed no significant differences in the characteristics of participants (i.g., parent and children age, parental BMI, children BMI z scores, parent and children MVPA, parent and children sedentary time, and parent and children mean of prolonged sedentary bouts) between the excluded and included samples (all *p*-values > 0.05). Table 1 shows the participants and family characteristics of included and excluded samples. The final study sample consisted of 30 parent and child dyads. Most parents were female (*n* = 21, 70%). Twenty-seven parents (90%) had at least a bachelor’s degree. The majority of parents (*n* = 29, 96.6%) reported a family income range between SAR 10,000 and 19,000, which was classified as middle. Twenty-four (80%) children in the current study reported one or more children in the household.

### 2.2. Study Context

In this study, data were collected from July to September 2020. At the time of data collection, the Saudi government began easing the pandemic restrictions with special rules. For example, people were able to go outside of their home for exercise and shopping. However, restrictions such as social distancing, self-quarantine, and closure of schools have continued. Restrictions on social gatherings of more than 50 people have also remained unchanged.

### 2.3. Children and Parents’ Body Mass Index (BMI) Measures

All measurements were conducted in a sports court at the Leader’s Preparation Institute of Riyadh. Parents and their children’s height and body mass were measured individually to the nearest ±0.1 cm and ±0.1 kg, respectively. The researchers recorded all measurements while the participants were wearing light clothing and no shoes. Body mass index (BMI) was calculated by dividing participant weight by height squared [weight (kg)/height^2^ (m^2^)], and converted to z scores using the LMS method [31]. According to the International Obesity Task Force (IOTF) criteria [32], children’s BMI was classified as normal weight, overweight, or obese. Parents’ BMI was classified into normal weight (18.5 to 24.9 kg/m^2^), overweight (25.0 to 29.9 kg/m^2^), and obese (≥30.0 kg/m^2^).

### 2.4. PA and Sedentary Measures

The ActiGraph 3-axis accelerometer (wGT3X-BT, ActiGraph LLC, Pensacola, FL, USA) was attached to the parents’ and their children’s right hip using an elastic waistband and data were collected for 24 h across 7 days. The participants were instructed to remove the device for water-based activities (i.e., bathing, showing, and swimming). Accelerometer data were initialized, downloaded, and processed using ActiLife software (version v6013.3, ActiGraph LLC, Pensacola, FL, USA). The accelerometer was set up to record the data at a sampling rate of 30 Hz. Data were downloaded as a raw format and converted to the AGD format using 60 s for children and parents. For the purpose of this study, only waking time was analyzed. The non-wear time was determined to be 20 min or more of consecutive zeros during the waking time [33]. The waking time was determined for each participant using daily log and graphic data displayed in the ActiLife software. For children’s data, established accelerometer cut-off points based on the vector magnitude (VM) method were used to determine sedentary and MVPA intensities as counts per minute (CPM): sedentary ≤ 720 CPM, LPA 721–3027 CPM, and MVPA ≥ 3028 CPM [34]. For the parent, accelerometer cut-off points based on VM were also used to classify parents’ sedentary time [35] and MVPA [36]; sedentary: <150 CPM, LPA 151–2689 CPM, and MVPA ≥ 2690 CPM. Consistent with previous research, children and parents’ total number of prolonged sedentary bouts of ≥20 min with no tolerance and number of breaks in sedentary time were calculated [37,38]. Breaks in sedentary time were defined as any interruption of sedentary bouts by ≥1 min of light or MVPA [37,39]. Accelerometer data were considered valid if the participants wore the accelerometers for at least 600 min/day and for a minimum of 3 days [40].

### 2.5. Data Analysis

SPSS 26.0 (IBM, Chicago, IL, USA) was used for all statistical analyses. The data are reported as mean and standard deviation (±SD) for normally distributed data and median and (25th and 75th) percentiles for skewed data. Descriptive data were calculated for males and females and for the entire sample. Significant differences between males and females (i.e., children and parents) and between included and excluded samples were evaluated using an independent *t*-test for normally distributed data and the Mann–Whitney *U* test for skewed data. Pearson correlations were used to examine the relationship between children’s and parents’ sedentary behaviors and MVPA. The relationship between sedentary behavior and PA in parents and children was investigated using multiple linear regression models with the enter method. Two separate multiple linear regression models were created to predict children’s MVPA and sedentary time. The dependent variables were children’s MVPA and sedentary time, while the predictors variables were parents’ MVPA and sedentary time. The initial model (unadjusted) was created and then each model was adjusted for accelerometer wear time. Additional covariates (i.e., parents and children gender, children’s BMI-z, parents’ BMI, parents and children age, presence of siblings in the household, house type, and parents and children and parents’ education levels, and family income) were included in all models. Covariates with *p*-value ≥ 0.2 were not included in the final models. The interaction between the gender of the child or the gender of the parent and the parent’s exposure variables was separately added into the models to determine their association with child’s PA and sedentary behavior. The preliminary results showed that the interactions did not contribute to any of the models, so we decided not to stratify the models by the gender of parents and children. Influential outliers were defined using Cocks distance >1. Variance inflation factors (VIFs) were used to assess multicollinearity among predictors and covariates variables. The preliminary analyses showed that the multicollinearity had no effect as all values were <10 [41]. Breusch–Pagan and Koenker tests were used to test the homogeneity of variance [42]. All *p*-values were ≥ 0.05 indicating the presence of homoscedasticity. Statistical significance was set at alpha < 0.05.

## 3. Results

Table 2 shows the characteristics of the final sample, stratified by gender. Among the parents, 73.3% were overweight or obese. Overweight or obese children accounted for 20% of the sample. Children’s average accelerometer wear time was 766.1 min/day, with 90% of children providing at least four days of wearing time. A total of 86% of parents provided at least four days of accelerometer wearing time, with an average of 826.3 min/day. Children and parents spent about 51.3% and 57.3% of their wear time sedentary, respectively. Parents spent about 4.3% of their time in MVPA, whereas children spent about 8.6% of their time in MVPA. 

Our findings revealed no significant differences in anthropometric measurements, sedentary behaviors, or MVPA levels between the boys and girls (all *p*-values > 0.05). However, compared with female parents, our data showed that male parents were taller (10.7; 95%CI 5.8, 15.6; *p* = 0.001) and accrued more MVPA time (21.4; 95%CI 6.3, 36.5; *p* = 0.007) (Table 2). Our data showed that 50% of children and 53% of parents met the recommended amount of PA in Saudi Arabia (Table 2). Our data also showed that 100% of the children’s sample exceeded the Saudi recommended amount (<2 h/day) of sedentary time.

The outcomes of bivariate correlations between accelerometer-measured child and parent sedentary time and MVPA are presented in Table 3. The data shows that child sedentary time was positively correlated with parent sedentary time in the entire sample (r = 0.477, *p* = 0.008). In contrast, child MVPA was negatively correlated with parent MVPA (r= −0.400, *p* = 0.029).

The outcomes of the linear regression models of the association between parents’ and children’s’ sedentary time and MVPA are shown in Table 4. The unadjusted model (Model 1) revealed that there was a significant positive relationship between sedentary time of parents and children (*B* = 0.425, *p* = 0.008). When adjusting for wear time, age of child, and house type (Model 2) there was no longer a significant association between parents’ and children’s sedentary time. The unadjusted model (Model 1) showed that children’s MVPA was negatively associated with that of their parents. This negative association remained significant and became stronger when adjusted for wear time, child age, and house type (Model 2).

## 4. Discussion

This is the first study to objectively measure PA and sedentary behavior in Saudi primary school children and their parents. Additionally, our study is novel because it objectively examined the association of children’s physical activity and sedentary time with parents’ physical activity and sedentary time during the COVID-19 pandemic. According to the findings, Saudi children and their parents spent 8.6% and 4.3% of their wear time in MVPA, respectively. This study also showed that both children and their parents spent more than 50% of accelerometer wearing time sedentary. The results presented in this study suggest a negative relationship between children and their parents with respect to the MVPA in all models during the COVID-19 pandemic. Unadjusted data showed that a significant and positive relationship was discovered between children and their parents’ sedentary behavior.

### 4.1. Children’s Time Spent in PA and Sedentary Behaviors during the COVID-19 Pandemic

The mean estimate of children’s MVPA (65 min/day) in our study was surprising and contrary to previous device-based measures that have reported lower MVPA among children during the COVID-19 pandemic. For example, Dallolio et al. [43] found that Italian primary school children spent less time (40 m/d) on MVPA. Ten Velde et al. [44] reported a lower MVPA time (48 m/day) in 7–10-year-old children in the Netherlands. A recent systematic review concluded that among 84 studies most (*n* = 57) reported a decline in PA during the COVID-19 pandemic [13]. However, few previous researchers have found an increase in MVPA in children during the COVID-19 pandemic, which is consistent with our findings. For example, Alonso-Martinez et al. [45] reported that Spanish preschool children spent 74 m/day in MVPA. Other studies have reported an increase in MVPA, reaching 105 min/day [15] and 124 min/day [16]. Our data also showed that, during the pandemic, 50% of children in this study met the Saudi MVPA guidelines. This proportion is higher than that recently reported in a parent-based survey study. Alanazi et al. [12] reported that during the COVID-19 pandemic approximately 36% of Saudi children met the MVPA guidelines. The high level of MVPA and the increase in the number of children meeting the MVPA guidelines reported in our study can be attributed to factors related to the living environment. For example, most children in our study lived in houses with outdoor spaces. Thus, it is likely that having more home space can be a factor increasing PA in children. Pombo et al. [46] suggested that the presence of an outdoor space with a home compound may positively influence PA in children. It has also been reported that house play space was positively associated with children’s MVPA [47]. Another factor that may have positively influenced children’s MVPA in our study was having siblings in the household. A total of 80% percent of children in the current study have at least one brother or sister. A systematic review and meta-analysis suggested that children living with siblings spent more time in MVPA than other children [48]. 

With regards to children’s sedentary time, our data showed that time spent sedentary was lower compared with earlier research studies from the Netherlands [44], Hong Kong [15], and Spain [45] where 465, 601, and 659 min/day were reported, respectively. A recent systematic analysis of research that employed questionnaires to report sedentary time likewise found an increase in sedentary time [49]. The results of this study also showed that during the pandemic 100% of children in this study exceeded the Saudi sedentary guidelines. This proportion is higher than that recently reported in a parent-based survey study [12]. Previous data showed that, during the pandemic, approximately 85% of Saudi children exceeded the recommended amount (<2 h/day) of sedentary time [12]. The higher proportion of children that exceeded the sedentary recommendation in our study can be attributed to many reasons. For example, compared with previous data, our data were collected during the summer when schools were closed. It has been reported that children tend to spent more time sedentary during summer holidays [50]. Another reason can be the increased number of children with electronic devices during the pandemic. Recently, Saudi data showed that during the pandemic the proportion of children having electronic devices increased from 40% to 70% [51]. 

Differences in MVPA and sedentary time compared with earlier research can be attributed to country-specific restrictions during the COVID-19 pandemic. In this research, the data were collected in the summer of 2020, when most of the COVID-19 restrictions in Saudi Arabia were relaxed. Thus, families and children were able to go outside homes where non-organized activities were allowed. Differences with earlier studies might also be methodologically attributed to the use of different monitors, cut-off points, and epochs. For example, in this study, the cut-off point was developed based on vector magnitude data, whereas in previous studies, cut-off points based on a vertical axis were employed. It has been shown that varying cut-off points have a significant impact on the estimated time spent in PA and sedentary behavior [52].

### 4.2. Parents’ Time Spent in PA and Sedentary Behaviors during the COVID-19 Pandemic

We found that the parents in our study had higher levels of MVPA on a daily basis. This is supported by the findings of previous device-based studies from the USA and UK. For example, data from the USA showed that adults accumulated 40, 42, and 44 min/day in MPVA during March, April, and May 2020, respectively [53]. These amounts of MVPA are higher than what we found in our study (34.6 min/day). In the same line, during the pandemic lockdown, data from the UK showed an increase by an average of 11.7 and 20.5 min in adults’ MVPA [54]. Data from Spain also showed that 74% of the study sample (*n* = 20) met the adult recommended PA [55]. Similar to our results, a recent Saudi data showed that 54% adults engaged in daily MVPA [56]. Contrary to our findings, national survey data showed a reduction in PA levels among Saudi adults [18], and 80.6% of Saudi adults have not achieved the PA recommendation [57]. With regards to adults’ sedentary time, a recent systemic review research showed that, during the COVID-19 pandemic, a significant increase in adults’ sedentary time was found among most of the previous studies [49]. National data from a self-report study showed a large increase in sedentary time in Saudi adults (men = 9.5 h/day and women = 11.6 h/day) than that we found [57]. However, our results are in agreement with survey data which showed that, on average, Saudi adults spent 7.7 h/day sedentary [56]. The COVID-19 restrictions alongside screen and social media time may have significantly contributed to increasing sedentary time in Saudi adults. For example, an additional 60 min in daily sedentary time has been reported in adults who strictly followed the COVID-19 guidelines compared with those who did not [58]. Additionally, recent data showed that screen and social media time significantly increased by 20.5% and 18.7%, respectively [59]. However, it should be noted that the parents in our study were young (mean age 38.9 years) and 70% were females. During the COVID-19 pandemic, it was reported that younger adults engaged in more sedentary time [60], and female had less levels of PA [61]. Our study supports the previous findings that females engaged in less PA levels compared with males [61,62,63]. Although, the pandemic restrictions provided less opportunities to engage in PA for both gender, females were had more barriers to participate in PA [63]. 

### 4.3. Child and Parent Association of PA and Sedentary Behaviors

The findings of this study revealed a negative relationship between MVPA in children and MVPA in their parents. These results are contrary to those of two previous studies which used accelerometers and showed a positive association between pre-pandemic children’s MVPA and their parents’ MVPA [28,29], but not when they were apart [29]. Comparing our findings with the results of earlier research, which also used an accelerometer to measure parent–child PA and sedentary time, is difficult. This is due to differences in sample characteristics (data were recorded in one or both parents), age range across studies, and different methods used to analyze PA and sedentary time. The contradictory results with previous studies can also be explained by their research context, since they were focusing on parental support of children PA and co-participation among parents and children.

Our findings are similar to those that reported a negative association [64,65,66,67], no association with male parents [68], and no association at all [69] between children and parents’ MVPA during the pre-pandemic period. This study had no data to identify whether parents and their children were present during the activity time. Thus, the negative association found in this study can be explained by the fact that parents and children did not spend more time doing activities together. However, a previous study reported that children accumulated more accelerometer counts and less sedentary time when they participated in activities with their parents [70]. Additionally, the relationship between parent and child PA was found to be stronger when they are together than when they are apart [24,65,66,71]. The negative association found in this study can also be explained by the presence of other children in the household or by peer role modeling rather than parent role modeling. It has been suggested that friends might influence children’s PA more than parents [72]. Another explanation can be that parents may spend more time in sustained and/or vigorous activities such as jogging, swimming, or gym exercises that young children cannot perform [66,67]. A higher proportion of male parent MVPA can be another reason for the negative association between children and parent MVPA. In our study, male parents spent an average of 49 min/day of MVPA. Barkin et al. [66] reported an inverse association between child and parent MVPA when parental MVPA exceeded 40 min/day. Furthermore, the detrimental relationship discovered in this study can be explained by the parents’ characteristics. Compared with children living with employed parents, a self-reported study revealed that Saudi children were more physically active when their parents were unemployed [73]. However, because of the negative connection between parent and child MVPA, interventions focusing on a single group may not be appropriate to promote MVPA at this age. 

In the unadjusted model, the findings of this study showed that children’s sedentary time was positively associated with parents’ sedentary time. The significant association was no longer evident when the model adjusted for covariate variables. The non-significant association between parent and child sedentary time, reported in this study, may be a result of the small sample size. However, our findings are in line with previous studies that reported that parents’ total sedentary time was not associated with their adolescents’ total sedentary time [74]. Another study found that the association between parent and child sedentary time was weaker when apart [24]. However, our findings did not support previous studies that reported a positive association of parental sedentary behavior in children [66,75]. Thus, our data suggest that children’s sedentary behavior may be influenced by factors other than their parents’ sedentary behavior. For example, in this study, we did not examine the association between parents and children’s sedentary time during a specific period of time (i.e., morning vs. afternoon or weekdays vs. weekends). A previous study reported a strong association between mothers and their preschool children’s sedentary time in the morning compared with the afternoon and evening [76]. Our results showed that accelerometer wear time and house type adjusted the association between parents’ and children’s sedentary time. Thus, this type of study should ensure that results are adjusted for accelerometer wear time and house type. 

### 4.4. Strength, Limitations, and Implications

The novelty of our study, among other studies conducted during the COVID-19 pandemic, is the use device-based measures to examine the association between children and their parents’ PA and sedentary behaviors during the COVID-19 pandemic. Further, this study may be the first to objectively measure PA and sedentary behavior within a family context in Arabic countries. However, the results of the current study should be interpreted with caution. It should be noted that we experienced a poor response to participate in the current study which affected the amount of the required sample size. This was not surprising given the challenges of conducting device-based measurement for the first time in the family context in Saudi Arabia and during the COVID-19 pandemic. This is a cross-sectional design, so we do not know how active the participants were before the pandemic. Thus, we are unable to define how the pandemic restrictions affected our sample PA and sedentary behaviors. The small sample size, and that 70% of the parents were mothers, may affect the generalization of the findings. Thus, future work should investigate whether our results are different or consistent with large samples and samples of fathers. We excluded 19% of the study sample due to noncompliance, reducing the sample size which may increase the possibility of bias. However, this possible bias is likely to be minimal due to the lack of significant differences in variables between those included and excluded. Another limitation of this study is the use of a convenience sample, which is prone to selection bias. Twenty-seven parents (90% of study sample) had at least a bachelor’s degree, and the majority of parents (*n* = 29, 96.6%) reported a family income range between SAR 10,000 and 19,000. These levels may not represent the national norms of education levels and family income and may have an effect on the participants’ PA and sedentary behaviors. For further research to estimate the PA levels and sedentary time across different populations it would be beneficial to identify populations who need targeted interventions. In this study, the presence of a parent with children was not reported. This kind of information would be useful in designing intervention programs for promoting PA in children and parents. In Saudi Arabia, there is no objective data that allow us to compare our data. Thus, future cross-sectional and longitudinal studies are needed to continue exploring children and parents’ PA and sedentary behaviors. Finally, the use of different accelerometer cut-off points for children and parents may influence the association between children’s and their parents’ PA and sedentary time. It has been reported that the selection of accelerometers’ cut-off point may influence in estimating PA intensities [77].

Our study has several implications and suggestions for policymakers, healthcare providers, researchers, practitioners, and parents. Policymakers and healthcare providers should first consider the short and long-term health effect of developing restrictions on children and parents’ behaviors during the pandemic. For researchers and practitioners, targeted intervention to support family healthy behaviors and prevent health risk, specifically with respect to sedentary behaviors is needed. The available data of the association between children and their parents’ behaviors suggest that family context might be a useful strategy for changing behaviors. For parents, additional efforts are required to offer PA opportunities and reducing sedentary time, especially for children with no outdoor space available. 

## 5. Conclusions

The findings of the current study found that 50% of children and 53% of parents had sufficient levels of MVPA to meet the PA guidelines. Children in this study exceeded the Saudi recommended amount (<2 h/day) of sedentary time. Thus, future work needs to investigate the related correlates for better understanding this behavior and to create targeted intervention. Our results support previous device-based studies that have reported an increase in children and parents’ MVPA and sedentary time during the pandemic. Children’s MVPA and sedentary time were both correlated with that of their parents. Adjusted linear regression showed only child MVPA was negatively associated with their parents’ MVPA. There is evidence that multi-level interventions involving parents and children are more effective than interventions focusing on a single group. This study also provides evidence to support the link between MVPA and sedentary time between parents and children.

## Figures and Tables

**Table 1:** Description of participants and family characteristics.

	Included Sample	Excluded Sample
**Factor**	*N* (%)	*N* (%)
**Children gender**		
Boys	14 (46.7)	3 (42.8)
Girls	16 (53.3)	4 (57.2)
**Parent gender**		
Male	9 (30)	2 (28.6)
Female	21 (70)	5 (71.4)
**Parent education level**		
Undergraduate	27 (90)	6 (85.7)
Postgraduate	3 (10)	1 (14.3)
**Family income**		
Low (<10,000 SR)	-	-
Middle (SAR 10,000–20,000)	29 (96.7)	6 (85.7)
High (SAR > 20,000)	1 (3.3)	1 (14.3)
**Home type**		
Home with play space	18 (60)	5 (71.4)
Home without play space	12 (40)	2 (28.6)
**Siblings**		
No	6 (20)	1 (14.3)
One or more	24 (80)	6 (85.7)

**Table 2:** Descriptive statistics for the study variables across groups that are stratified by gender.

Characteristics	Children			Parents		
	Total Sample	Male	Female	Total Sample	Male	Female
	*n* = 30	*n* = 14	*n* = 16	*n* = 30	*n* = 9	*n* = 21
**Age (years)**	9.1 (1.8)	9.2 (1.9)	9.0 (1.8)	38.9 (7.6)	39.6 (9.6)	38.6 (6.8)
**Height (cm)**	130.0 (12.6)	128.7 (11.5)	131.7 (13.7)	161.0 (7.7)	168.7 (7.5) *	158.0 (5.3)
**Weight (cm)**	30.4 (9.4)	29.6 (9.5)	31.1 (9.7)	70.1 (11.4)	76.4 (10.0)	68.0 (11.3)
**BMI (kg/m^2^)**	18.1 (5.0)	17.5 (4.2)	18.6 (5.8)	27.2 (4.3)	26.8 (3.4)	27.3 (4.7)
**BMI-z**	0.3 (1.3)	0.3 (1.2)	0.4 (1.2)	-	-	-
**MVPA (min/day)**	65.6 (35.1)	70.2 (43.2)	61.7 (27.1)	34.6 (20.7)	49.6 (19.4) *	28.1 (18.1)
**Sedentary time (min/day)**	442.2 (121.8)	481.3 (141.1)	408.0 (93.6)	427.9 (136.7)	471.7 (170.0)	409.2 (119.6)
**Meeting PA Guidelines (*N*)%**	15 (50)	9 (64.2)	6 (37.5)	16 (53.3)	6 (66.7)	10 (47.6)
**Sedentary bouts ≥20 min (min/day)**	31.7 (4.1)	32.2 (4.8)	31.4 (3.4)	29.7 (3.4)	31.3 (4.5)	29.0 (2.6)
**Sedentary bouts (number/day)**	2.7 (2.0, 5.2)	2.3 (1.4, 8.5)	3.4 (2.0, 4.7)	2.2 (1.3, 4.3)	3.4 (1.8, 5.0)	2.2 (1.2, 3.7)
**Break in sedentary (number/day)**	76.0 (16.4)	75 (15.3)	76.8 (17.7)	91.4 (25.4)	101 (26.0)	87.0 (24.5)
**Accelerometer wear time (min/day)**	766.1 (101.6)	792.8 (105.8)	742.5 (94.8)	826.3 (141.7)	886.1 (141.5)	800.0 (137.2)

MVPA: moderate and vigorous physical activity; BMI: body mass index; Data presented as mean (±SD) or median (25th, 75th); * significantly different as compared with female parents.

**Table 3:** Pearson correlation between parents’ and children’s PA and sedentary behaviors.

	Child Sedentary (min/day)		Child MVPA (min/day)	
	r	*p*	r	*p*
Parent sedentary (min/day)	**0.477** *	0.008	−0.009	0.963
Parent MVPA (min/day)	0.283	0.130	−**0.400** *	0.029

MVPA: Moderate and vigorous physical activity; * *p* < 0.05.

**Table 4:** Linear regression for the association between children’s and parents’ PA and sedentary behaviors.

	Model 1 (Unadjusted)			Model 2 (Adjusted)		
Exposure	B (95% CI)	SE	*p*	B (95% CI)	SE	*p*
Parent sedentary time (min/day)	**0.425** (**0.122**, **0.728**) *	0.148	**0.008**	0.009 (−0.352, 0.370)	0.175	0.959
	R^2^ = 0.22; Adjusted R^2^ = 0.20			R^2^ = 0.54; Adjusted R^2^ = 0.45		
Parent MVPA (min/day)	−**0.677** (−**1.277**, −**0.076**) *	0.293	**0.029**	−**0.828** (−**1.320**, −**0.336**) *	0.238	**0.002**
	R^2^ = 0.16; Adjusted R^2^ = 0.13			R^2^ = 0.54; Adjusted R^2^ = 0.44		

Child MVPA and sedentary behaviors were the outcome variables; all models were adjusted for children’s and parents’ accelerometer wear time, children’s age, and house type; * *p* < 0.05.

## Data Availability

All the relevant data are available in the manuscript.
